# Rapamycin ameliorates chitosan nanoparticle-induced developmental defects of preimplantation embryos in mice

**DOI:** 10.18632/oncotarget.10813

**Published:** 2016-07-24

**Authors:** Yun-Jung Choi, Sangiliyandi Gurunathan, DaSom Kim, Hyung Seok Jang, Woo-Jin Park, Ssang-Goo Cho, Chankyu Park, Hyuk Song, Han Geuk Seo, Jin-Hoi Kim

**Affiliations:** ^1^ Department of Stem Cell and Regenerative Biotechnology, Humanized Pig Research Center (SRC), Konkuk University, Seoul, Republic Korea; ^2^ Department of Pathology, Hanyang University Medical Center, Wangsimni-ro, Seongdong-gu, Seoul, Republic of Korea

**Keywords:** chitosan nanoparticles, Rapamycin, preimplantation, autophagy, ER stress

## Abstract

Chitosan nanoparticles (CSNPs) are used as drug or gene delivery vehicles. However, a detailed understanding of the effects of CSNPs on embryonic development remains obscure. Here, we show that CSNPs can be internalized into mouse blastocysts, such as the zona pellucida, the perivitelline space, and the cytoplasm. Consequently, CSNPs-induced endoplasmic reticulum (ER) stress increases both of Bip/Grp78, Chop, Atf4, Perk, and Ire1a mRNAs expression levels, and reactive oxygen species. Moreover, CSNPs show double- and multi-membraned autophagic vesicles, and lead to cell death of blastocoels. Conversely, treatment with rapamycin, which plays an important role as a central regulator of cellular proliferation and stress responses, decreased CSNPs-induced mitochondrial Ca^+2^ overloading, apoptosis, oxidative stress, ER stress, and autophagy. In vivo studies demonstrated that CSNPs injection has significant toxic effect on primordial and developing follicles. Notably, rapamycin rescued oxidative stress-induced embryonic defects via modulating gene expression of sirtuin and mammalian target of rapamycin. Interestingly, CSNPs treatment alters epigenetic reprogramming in mouse embryos. Overall*, these observations suggest* that rapamycin treatment could ameliorate CSNPs-induced developmental defects in preimplantation embryos. The data from this study would facilitate to understand the toxicity of these CSNPs, and enable the engineering of safer nanomaterials for therapeutic applications.

## INTRODUCTION

Nanotechnology is a key technology that plays an important role in various disciplines, such as electronics, the optical industry, environmental engineering, biotechnology, and nanomedicines [1–4]. It is very well known that chitosan nanoparticles (CSNPs) are non-toxic, biodegradable polycationic polymers with low immunogenicity [5]. However, previous studies have shown that CSNPs are cytotoxic to tumor cell lines [6]. Furthermore, Loh et al. [7] found that CSNPs could be internalized by human liver cells, reducing the cell's viability and proliferation and compromising the integrity of the cell membrane.

Titanium dioxide nanoparticles (TiO_2_ NPs) can accumulate in the ovaries, and this accumulation results in ovarian damage, causes an imbalance in the distribution of mineral elements and sex hormones, decreases fertility, and causes oxidative stress in mice [8]. The daily inhalation of cadmium oxide nanoparticles (CdO NPs, 230μg/m^3^) increased the uterine weight and altered the placental weight of pregnant CD-1 mice [9]. At a dose of 0.8 mg/mouse in pregnant mice, silica nanoparticles (SiO_2_) and TiO_2_ NPs caused a decrease in the uterine weight and an increase in the fetal reabsorption rate [10]. Many NPs, such as TiO_2_ NPs, SiO_2_ NPs [10], quantum dots (QDs) [11], and carbon NPs [12], can also penetrate the placental barrier. Exposure to NPs during the gestational period affects fetal organogenesis and morphology [13]. Several NPs exhibit detrimental effects on both male and female fertility and fetal development, and these adverse effects are related to NP composition, surface modification, dose, exposure route, and animal species [14].

Besides metal nanoparticles, non-metallic nanoparticles also have influence in toxicity of reproductive systems. For example, Farombi et al. [15] showed that intraperitoneally administered carbon nanotubes (CNTs) in rats at a dose of 0.25~1.0 mg/kg for 5 days produced marked histopathological changes in both the testis and epididymis, decreased the plasma testosterone levels, and increased oxidative stress in both the testis and spermatozoa. The effects of CNTs on embryonic development in CD1 female mice were observed through CNTs injections on gestational day (GD) 5.5. They found that CNTs induced early miscarriages, fetal malformations, and oxidative stress in malformed fetuses [16]. Recently, Xu et al. [17] studied the effects of reduced graphene oxide (RGO) on female mouse reproductive ability, at 6.25-25 mg/kg one or 30 day before mating or on gestation day 6 or 20. The results suggest that RGOs has no significant reproductive adverse effects; however, when small-RGO was administered during late gestation, most of the pregnant mice died and some mice had malformed fetuses. In another study, Tsuchiya et al. [13] injected the pregnant mice with fullerene at doses of 25-137 mg/kg on GD10 and GD11, and demonstrated that most of the embryos were died after 18 h of treatment. Among them, 50% of the embryos showed morphological abnormalities at the 50 mg/kg dose, especially in head region. Particularly, treatment with 50 mg/kg had a seriously harmful effect on the yolk sac, such as a shrunken membrane and narrowed blood vessels.

In our previous study, we showed that after morula stage embryos were developed to blastocyst in culture media with or without CSNPs, when they were transferred to recipients, the percentage of blastocysts resulting in viable pups was significantly reduced. These detrimental effects are linked to reduced total cell numbers, enhanced apoptosis, and abnormal blastocoel formation at the blastocyst stage, indicating that CSNPs treatment might have long-term adverse biological effects during pregnancy [18]. The unique physicochemical properties of CSNPs can enhance their biological effects, and they could cause developmental toxicity to embryos. There are growing concerns regarding the effects of NPs on pregnant women and the possibility that they can cross the placental barrier and cause fetal harm [13, 19–23]. However, little is known regarding the potential toxicity of CSNPs on embryo development in the context of animal studies. This area of study is important because CSNPs are administered to the human body *via* several routes to various tissues through the systemic circulation [24–26].

Rapamycin plays an important role as a central regulator of metabolism, growth, cellular proliferation, stress responses, and cell cycle progression [27] and the dysregulation of mTOR is linked to the development of various chronic diseases including insulin resistance, diabetes, cardiovascular disease, and obesity; as well as progression of various types of cancer [28–30]. Previous study suggested that rapamycin can protect the neuronal cells from apoptosis induced by rotenone treatment by inducing autophagy [31].

In this study, firstly we investigated the mechanism of CSNPs-induced embryo damage caused by oxidative and ER stress and autophagy. Secondly, we evaluated the protective effect of rapamycin on CSNPs-induced damage of preimplantation embryos and finally, we investigated short-term *in vivo* toxic effects of CSNPs on ovary, embryo development, and implantation in female mice.

## RESULTS AND DISCUSSION

### Cellular uptake and effects of CSNPs on blastocyst-stage embryos

A previous study reported that different types of cell death (apoptosis, necrosis, and autophagy) contribute to the pathophysiology of different human disorders [32], in which autophagy is one among process of cell death and autophagy acts as either a survival or death safeguard mechanism on different environmental stresses and cell types. Recent studies suggest that autophagy, a highly-regulated intracellular process for the degradation of long-lived proteins and damaged organelles, may represent a general cellular and tissue response to oxidative stress [33, 34]. However, when oxidative stress reaches a level beyond the control of cellular protective mechanisms, cell death will occur through necrosis, apoptosis, or autophagic cell death [35, 36]. In this study, we are interested to investigate whether CSNPs triggered the production of excessive intracellular reactive oxygen species (ROS), resulting in autophagic death of preimplantation embryo. We examined the interaction of CSNPs with preimplantation embryos and the activation of various genes involved in embryonic physiological processes. The embryos treated with CSNPs showed severely swollen mitochondria, autophagosomes (mitophagosomes), lipid droplets (lipophagy), and lysosomes were the predominant types of autophagic vacuoles (Figure S1), indicating that removing the damaged mitochondria and the accumulated lipid droplets is most pertinent for protecting host cells from CSNPs-induced injury.

**Figure 1:**
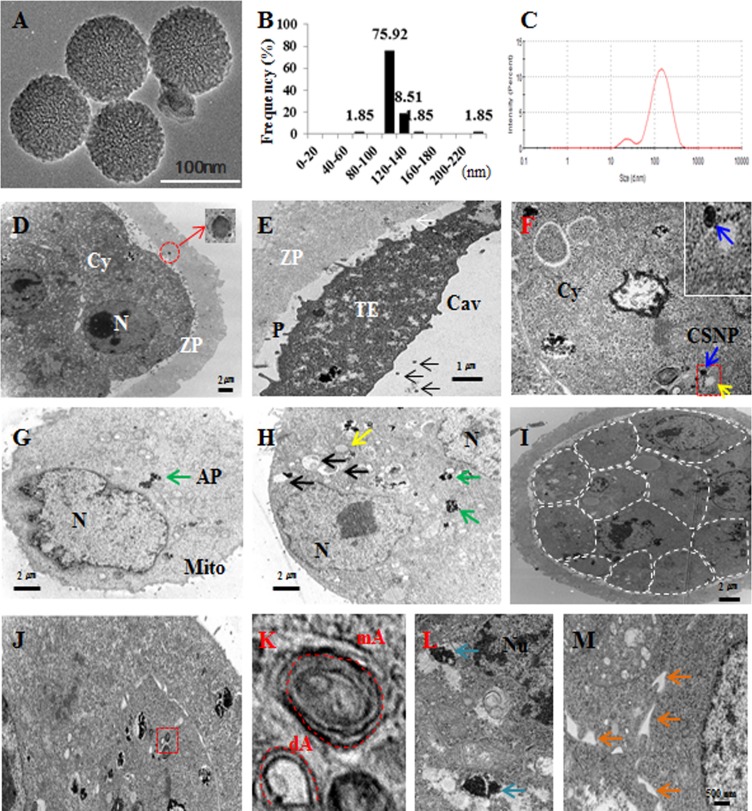
Characterization and localization of CSNPs **A.** TEM images of several fields were used to measure the particle sizes of CSNPs. **B.** Histogram showing the particle size distribution ranging from 80 nm to 160 nm based on TEM images of CSNPs. **C.** DLS of CSNPs. **D.** CSNPs penetrated the zona pellucida and exited inside the zona pellucida. The red circle indicates CSNP and shows high magnification. **E.** CSNPs passed the zona pellucida and are shown in the perivitelline space and cavity. **F**. Blue and yellow arrows indicate the CSNPs and autophagosomes. **G**. Green arrow indicates autophagosome (AP). **H**. Black, green, and yellow arrows indicate mitochondria, autophagosomes, and autophagice vacuoles, respectively. **I**. Dashed line showed each single cell of CSNPs treated blastocyst. **J**. The red box shows autophagy and autophagic vesicles. **K**. High magnification of J, dA and mA indicate double- and multi-membrane vesicles. **L.** Blue arrows indicate high magnification of autophagosome. **M**. Orange arrows indicate dilation of the ER lumen.

CSNPs are well suited for studying the influence of nanomaterials on various biological processes because they are more soluble in aqueous solutions than other nanomaterials [37]. We used CSNPs with a diameter of 100 nm to determine the CSNPs-induced damage on preimplantation embryos, according to our previous study [18]. The determination of the size of the NPs used is an important factor for assessments of toxicology, cellular distribution and uptake amount. Therefore, the size of the prepared CSNPs was determined using dynamic light scattering (DLS) and transmission electron microscopy (TEM). The synthesized CSNPs were distributed uniformly and showed spherical in shape (Figure 1A). The average size of the particles was 100 nm (Figure 1B) when it was estimated from measuring more than 200 particles from TEM images. From Dynamic Light Scattering (DLS) analysis, the average size was found to be 120 nm (Figure 1C), which is slightly larger than those observed in TEM, might be due to the influence of Brownian motion.

We next detected the uptake of CSNPs in blastocyst embryos using TEM to examine whether preimplantation embryos could internalize CSNPs. CSNPs have a large number of atoms, which enable its distinction from cellular structures using TEM. After treating morula-stage embryos with CSNPs (100μg/ml) for 24 h, CSNPs were found inside the zona pellucida and the perivitelline space (Figure 1D and 1E). CSNPs were numerous inside the cells and were dispersed in the cytosol (Figure 1F and 1G). Blastocysts showing the initial stages of apoptosis were filled with small and electron-lucent vesicles. The aggregated vesicles were frequently observed to amalgamate, followed by the appearance of “empty zones” in the cytoplasm, such as the formation of large vacuoles (Figure 1H), which were scarce in control embryos.

In the blastocysts, CSNPs mostly accumulated on and around autophagosomal membranes (Figure 1I and 1J). The autophagic vacuoles were recognized as double-membrane structures with contents ranging from granular cytoplasm, degenerated organelles and endoplasmic or protein aggregates. CSNPs-treated blastocysts showed double- and multi-membraned autophagic vesicles (Figure 1K and 1L; Figure S1). In addition, CSNPs caused the dilation ER membranes (Figure 1M). However, whether CSNPs entered the nucleus was somewhat unclear, even at the prolonged incubation time.

### CSNPs induce autophagy *via* ER-activated stress in blastocyst-stage embryos

**Figure 2:**
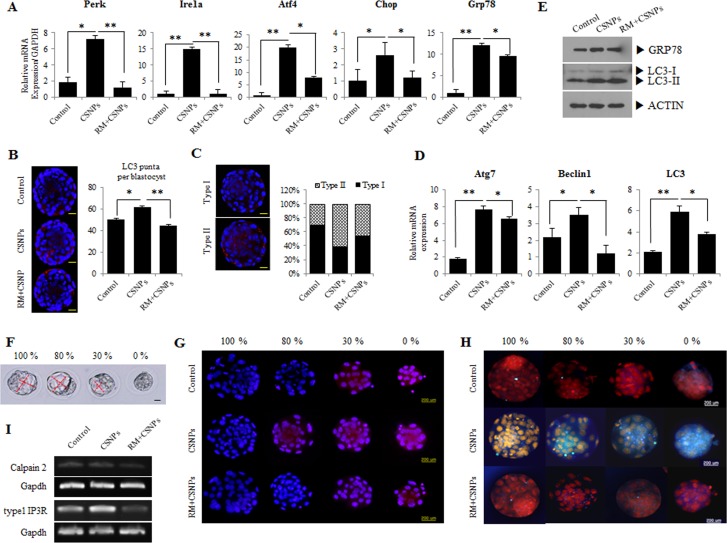
Effects of rapamycin on CSNPs-induced ER stress and autophagy in preimplantation embryos **A**. Morula embryos were cultured with CSNPs with or without rapamycin. Expression levels of genes involved in ER stress, such as Atf4, Ire1a, Chop, Perk, and Grp78, were measured by real time qRT-PCR. Rapamycin-treated groups restored their expression levels. **B**. Induction of autophagy by CNSPs and the rescue effect of rapamycin. Representative images of cells were taken using a fluorescence microscope. Note the red color showing the localization of punctate LC3. **C**. Based on immunostaining data, we divided the puncta into two groups (Types I and II). The numbers of LC3 puncta were counted and separated into 2 groups by expression patterns. **D**. The autophagy-related gene expression analyses were measured by real time qRT-PCR. Atg7, Belcin1, and LC3 mRNA expression levels were significantly induced in the CSNPs-treated group, but were restored by rapamycin treatment. **E**. Expression analysis of GRP78 and LC3 in CSNPs or rapamycin+CSNPs treated embryos using Western blot. **F**. CSNPs induced an abnormal developmental rate. Four groups were created based on the developmental rate. Ca^2+^ and ER stress in cells were analyzed. **G**. The effects of CSNPs and rapamycin on Rhod-2-AM fluorescence in blastocysts dependent on the developmental rate. **H**. Blastocysts from each group were labeled with ER tracker to visualize ER localization and expression. ER distribution/expression patterns were evaluated using fluorescence microscopy. **I**. Expression analysis of calpain2 and type 1 IP3R. * and ** indicate *p*<0.05,and *p*<0.01, respectively. RM indicates rapamycin.

Previous studies reported that ER stress can activate autophagy in plants and algae [38, 39], suggesting that the signaling pathways controlling autophagy activation are involved in response to this intracellular stress. To connect ER stress with autophagy, we are interested to study the link between ER stress and autophagy in CSNPs-treated embryos. Therefore, we examined the expression of ER stress-activated genes, such as Atf4, Ire1a, Chop, Perk, and Grp78/Bip. The expression levels of the ER stress-related genes were increased (Figure 2A). Thus, our observation demonstrated that CSNPs-induced autophagy required the classical autophagic machinery, including the autophagosome-initiating molecule Beclin 1, along with molecules involved in the membrane extension of the autophagosomal membranes: Atg7, and LC3 (Figure 2D).

Lysosomal turnover of the autophagosomal biomarker LC3-II reflects starvation-induced autophagic activity, and detecting LC3 by immunofluorescence has become a reliable method for monitoring autophagy and autophagy-related processes, including autophagic cell death [40]. In this study, we found that in an immunofluorescence assay, LC3-II expression and the number of puncta in blastocysts were noticeable in the CSNPs-treated groups, whereas the blastocysts pretreated with rapamycin and then treated with CSNPs had a lesser number of puncta than the CSNPs treatment group alone (Figure 2B). Interestingly, CSNPs-treated blastocysts showed 2 different patterns for LC3-II expression: the CSNPs-treated group had a higher ratio for the Type-II expression pattern than the expression pattern for Type-I as compared with the control or the CSNPs+ rapamycin-treated group (Figure 2C). The increased amount of cytoplasmic LC3^+^ signal per cell indicates that the conversion of LC3-I to LC3-II increased following a shift to CSNPs-supplemented medium and in response to rapamycin (Figure 2B and 2C). In contrast, LC3 punctate were less observed in control blastocysts. We propose that LC-3 expression most likely reflects the direct effects of CSNPs that are dependent on both apoptosis and autophagy machinery.

As shown in Figure 2B and 2C, the increased amount of cytoplasmic LC3^+^ signal per cell indicates that the conversion of LC3-I to LC3-II increased following a shift to both of CSNPs-supplemented medium and rapamycin+CSNPs: GRP78 and LC-3 protein expression in CSNPs-treated groups are significantly increased compared to control (Figure 2E). After the embryos were treated with CSNPs for 24 h, they showed different sized cavity, from 0 (arrested morula stage embryo) to 100% (normal stage blastocyst embryo) (Figure 2F). Because of the differences in the sizes of the blastocoels, it was apparent that different development levels were caused by CSNPs.

### CSNPs induces mitochondrial Ca^2+^ overloading

Ca^2+^ trafficking in and out of the ER regulate diverse cellular responses and signaling transduction pathways relevant to the stress response, the modulation of transcriptional processes, and development [41]. A study from Rizzuto et al. [41] suggested that the acute release of Ca^2+^ from the ER can trigger a variety of signaling mechanisms that promote cell death, mainly by Ca^2+^-mediated mitochondrial cell death. To assess the effect of CSNPs on the ER, we measured the ER stress level and the mitochondrial Ca^2+^ level in different-sized embryos with different blastocoel sizes (0%; arrested morula embryo, 30%, 80%, and 100%). As shown in Figure 2G and 2H, the treatment of mouse embryos with CSNPs significantly increased the staining intensity of ER-Tracker Blue-White DPX dye as compared with the control or the CSNPs- and rapamycin-treated groups. This observation suggested that rapamycin, as an ER stress inhibitor, can lead to the reduction of cellular injury in CSNPs-induced damage. These results indicate that the supplementation of the culture medium with CSNPs induces Ca^2+^ overloading at the mouse blastocyst stage. The above results implicate CSNPs as a kind of ER stress inducer that elicits unmanageable apoptosis in preimplantation embryos. Calpain proteins and inositol 1,4,5-triphosphate receptor type1 (IP3R1) are known to play important roles in various biological processes, including cell differentiation and apoptosis. As shown in Figure 2I, abrogating activities of Calpain2 and IP3R1 are closely implicated in the modulation of calcium levels by CSNPs in the ER.

### CSNPs induce ER stress and activate the UPR in mouse blastocyst-stage embryos

**Figure 3:**
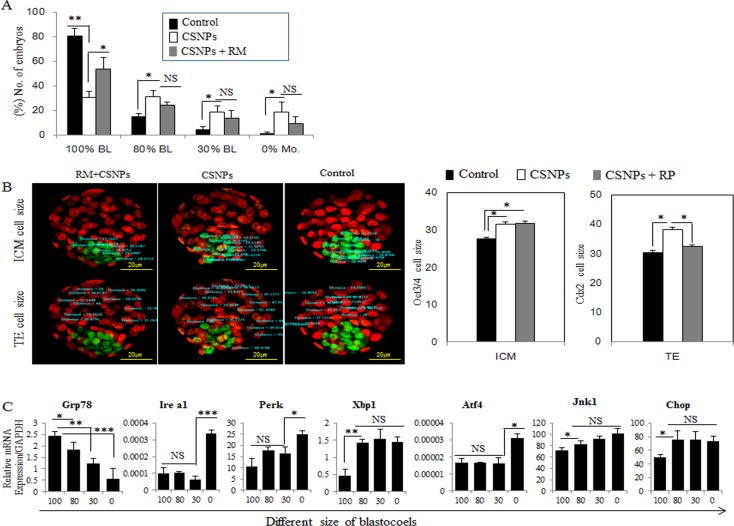
The rescue effect of rapamycin on CSNPs-induced ER stress **A**. After treatment with CSNPs, morula-stage embryos exhibited developmental delay. Blastocoel size was divided into four groups (0% to 100%) based on the effect caused by CSNPs. **B**. Immunostaining of OCT3/4 for the ICM marker and CDX2 for the trophoblast cell marker were performed, and then size was measured. The graph shows each cell size. The ICM cell size was larger when the ICM was treated with CSNPs and rapamycin compared to that of the control. In the TE, the cell size was recovered by rapamycin treatment. RP indicates rapamycin. **C**. The expression levels of various genes involved in ER stress, such as Grp78, Ire1a, Perk, Xbp1, Atf4, Jnk1, and Chop, were determined in each group of blastocysts. *, **, ***, and NS indicate *p*<0.05, *p*<0.01, p<0.001, and not significant, respectively..

The treatment of morula embryos with CSNPs revealed that the presence of these protein aggregates that are sometimes found inside autophagic vacuoles and ER dilation are characteristic of ER stress (Figure 1K, 1L, 1M and Figure S1). To validate this concept, we focused on the expression levels of ER stress- and unfolded protein response (UPR) marker-genes. Real time qRT-PCR analysis showed that Grp78/Bip, Ire1a, Perk, Atf4, and Chop/Gadd153 mRNAs expression levels in blastocysts treated with CSNPs were significantly increased compared with those of the control group (Figure 2A). It was necessary to determine the importance of ER stress and UPR markers in several stages of blastocyst development, including the following stages: no cavity (0%; arrested morula embryo), small cavity (30% blastocoel), middle-sized cavity (80% blastocoel) or 100% blastocoel. The rate of development for blastocysts with normal cavities in the rapamycin-treated group was significantly increased compared with those of the CSNPs-treated groups (Figure 3A). These observations demonstrated that pretreatment with rapamycin rescued the defects of the partially developed embryo through suppressing the ER stress-mediated response.

Relative sizes of the inner cell mass (ICM) and trophectoderm (TE) were determined using CDX2 and OCT3/4 staining. The cell size of the ICM was significantly increased in the group treated with CSNPs or/and the group treated with rapamycin+CSNPs as compared with the control. In the case of TE, the CSNPs-treated groups showed only a significant increasing in size compared with the control or the group treated with rapamycin+CSNPs (Figure 3B).

Therefore, we examined the expression of ER stress activated genes, such as Grp78/Bip, Ire1a, Perk, Xbp1, Atf4, Jnk1, and Chop/Gadd153. The expression levels of most of these genes were significantly higher in the 0% stage, except Grp78/Bip (Figure 3C). Among the members of the UPR signaling pathways, IRE1a is a key molecule that functions as a rheostat capable of regulating cell fate. IRE1a can stimulate the activation of apoptotic signaling kinase-1 (ASK1), which causes the downstream activation of the stress kinases Jun-N-terminal kinase (JNK) and p38 mitogen-activated protein kinase (MAPK), which promote apoptosis [42]. ATF6 is a transcriptional factor that, upon induction of ER stress, translocate to the Golgi compartment, where it is cleaved by the action of two proteases, Site-*2 protease* (S1P) and Site-*2 protease* (S2P). ATF4 drives the important targets a transcriptional factor C/Ebp homologous protein (CHOP), growth arrest, DNA damage-inducible 34 (GADD34), and ATF3 [43]. The activation of these ER stress genes may be responsible for CSNPs-induced cell death, and rapamycin can alleviate this CSNPs-induced ER stress. To obtain further evidence for the direct effects of the apoptosis of blastocysts treated with CSNPs, embryos were treated with CSNPs and the results showed that more than 50% of the CSNPs-treated embryos were arrested at an earlier stage of blastocyst development compared with the control group or the group treated with rapamycin+CSNPs (Figure 3A).

Next, we used different concentrations of rapamycin (5, 10, 50, 100μg/ml) to determine the optimal dosage of rapamycin that could prevent the CSNPs induced toxicity. As shown in Table S1, 100μg/ml of rapamycin significantly prevented CSNPs-induced embryo toxicity. These findings suggested that rapamycin had protective roles against CSNPs induced damages.

To determine whether CSNPs promote the survival or death of preimplantation mouse embryos, we exposed the embryos to autophagy activators, rapamycin and then evaluated the development of the CSNPs-treated embryos and their embryonic cell death (Figure 5A). Only the CSNPs-treated embryos showed significant detrimental effects in the embryo development process, whereas the embryos pre-treated with rapamycin had more developed blastocysts and fewer apoptotic cells. We determined that rapamycin+CSNPs treatment promotes embryo survival and protects against CSNPs-induced apoptosis. As shown in Figure 5B, mRNAs expression levels of Bax and Caspase-3 in CSNPs-treated embryos were significantly increased when compared with the control, whereas Bcl-2 mRNA expression was significantly decreased. Next, the total number of cells per blastocyst and the cell numbers of the ICM and TE were counted. The results suggest that the CSNPs-treated groups had significant reductions in the total numbers of cells when compared with the control group. Also, CSNPs-treated embryos had a small number of ICM, although after rapamycin treatment, the number of ICM was rescued (Figure 5C). Taken together, these results suggest that CSNPs treatment may promote apoptosis of blastocyst stage embryos.

### CSNPs induce mitochondria disorganization *via* the modulation of mTOR and Sirtuin signaling

**Figure 4:**
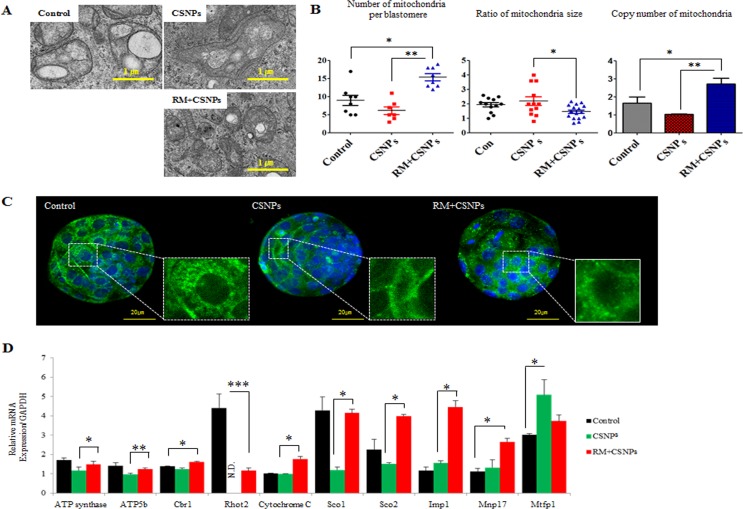
The rescue effect of rapamycin on CSNPs-induced mitochondrial damage **A.** Mitochondrial structural disorganization in blastomeres was determined by TEM. **B**. The number and size of mitochondria per blastomere were determined by TEN and copy numbers of mitochondria per blastomere were determined by real time qRT-PCR in the control group, the CSNPs-treated group and the group treated with both CSNPs and rapamycin..**C.** Mitochondrial morphology in the control group, the CSNPs-treated group and the group treated with both rapamycin and CSNPs was visualized *via* MitoTracker Green FM staining in different groups of blastocysts. **D.** Mitochondrial activity levels of ATP synthase, ATP5b, Cbr1, Rhot2, Cytochrome C, Sco1, Sco2, Imp1, Mvp17, and Mrfp1 were evaluated using real time qRT-PCR. *, **, ***, NS, and ND indicate *p* < 0.05, *p* < 0.01, *p* < 0.001, not significant, and not determined, respectively. RM indicates rapamycin.

CSNPs internalization causes structural disorganization in mitochondria. After internalization, the size of mitochondria was increased, however copy numbers of mitochondria per blastomere were reduced (Figure 4A and 4B). As shown in Figure 4C, CSNPs treated groups showed that mitochondria per blastomere in CSNPs-treated groups were significantly reduced compared to control group. Further, our results show that CSNPs influence the genes expression involved in mitochondrial activity, such as ATP synthase, Atp5b, Cbr1, Ras Homolog Family Member T2 (Rhot2), Cytochrome C, synthesis of Cytochrome C Oxidase 1 (Sco1), synthesis of Cytochrome C Oxidase2 (Sco2), Imp1, Myelin, lymphocyte protein 17 (Mvp17), and Mrfp1, whereas rapamycin rescues the defects caused by CSNPs (Figure 4D). Thus, these results suggest that CSNPs treatment may promote mitochondria disorganization of blastocyst stage embryos. To explore the possible mechanisms of mitochondrial dynamics impairment, we examined ROS level in blastocyst stage embryos at 12 h after CSNPs or rapamycin+CSNPs treatment. To provide evidence for CSNPs induce non radical ROS, we assessed CSNPs-induced ROS using the H2DCF-DA assay, which measures peroxide-dependent oxidation of H2DCF-DA to fluorescent DCF. As shown in Figure S2, the ROS signal level was five-folds higher in CSNPs-treated group than those of control or rapamycin+CSNPs treated group.

**Figure 5:**
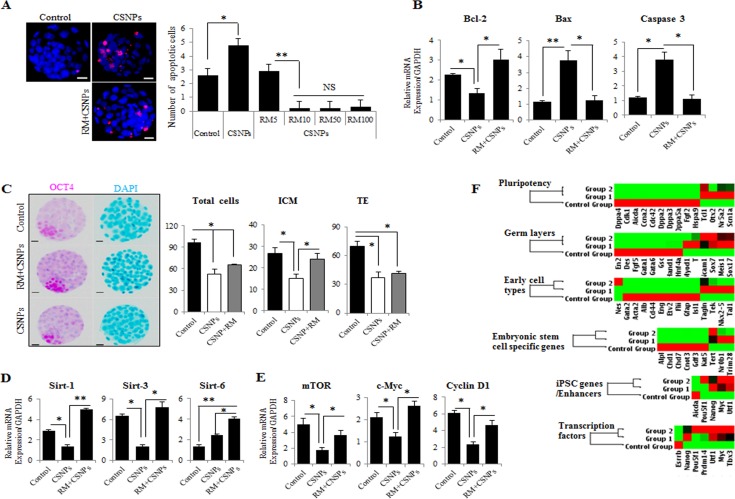
The effects of rapamycin on CSNPs-induced embryonic defects **A.** The determination of apoptosis in preimplantation embryos after CSNPs treatment. The numbers of apoptotic cells were measured using TUNEL-positive cells. Apoptotic cells were observed very rarely in the control group. The CSNPs-treated groups showed increased numbers of TUNEL-positive cells. After pre-treatment with rapamycin, apoptosis decreased. Various concentrations of rapamycin (5~100 μg/ml) resulted in the suppression of apoptotic cells. **B.** Real time qRT-PCR analysis was used to measure Bcl-2, Bax, and Caspase-3 mRNA expression. **C.** Immunostaining analysis of ICM and TE cells using CDX2 and OCT3/4. The total cell numbers were measured in the ICM and the TE. The CSNPs-treated group showed a lower number of ICM and a lower total cell number. Rapamycin rescued the effect of CSNPs. **D.** Real time qRT-PCR analysis of the Sirtuin family pathway. **E.** Real time qRT-PCR analysis of the mTOR pathway. **F.** The graphs of the validation of self-renewal, ICM, TE, and three germ cell layer markers. The heat map represents the changes in gene expression in the control, CSNPs-treated and rapamycin-pretreated embryos. The 6 groups are clustered around the horizontal axis with gene names on the vertical axis. *, **, and NS indicate *p* < 0.05, *p* < 0.01, and not significant, respectively. RM indicates rapamycin.

To determine the underlying mechanisms of mitochondrial defect and genomic stability caused by CSNPs, we examined Sirtuin family gene expression in CSNPs-induced embryonic cell death. As shown in Figure 5D, real time qRT-PCR results suggest that the expression levels of Sirtuins-1, and Sirtuins-3 mRNAs in the presence of CSNPs were markedly reduced (Figure 5D). However, rapamycin pretreatment increased Sirtuin-1, -3, and -6 mRNAs expression, recovered anti-apoptotic protein Bcl-2 mRNA expression, and reduced Caspase-3 mRNA expression (Figure 5B). As shown in Figure 2A, more than 50% of the CSNPs-treated embryos were arrested at an early stage of blastocyst development compared to the control group or the group treated with both of rapamycin+CSNPs. It is very well-known that SIRTUINS-1 expression has been reported to play crucial roles in the self-renewal and differentiation processes in response to environmental stress [44] or offset of aging process triggered by oxidative stress [45]. In addition, SIRTUINS-3 expression can also increase the expression of mitochondrial factors such as ATP synthase and Cytochrome C oxidase subunits [46] or can promote cell survival thorough deacetylation of Ku70, which involved in DNA repair, in response to genotoxic agents [47, 48]. Also, SIRTUINS-6 function has been associated with genomic stability, DNA repair, and gene silencing. It can deacetylate histones in H3K9Ac and contributing to heterochromatin formation and telomere stability [49]. Taken together, these observations indicated that down-regulation of Sirtuin-1 and -3 mRNAs expression leads to mitochondrial dysfunction *via* oxidative stress, which is one of the vital mechanisms for nanoparticle-mediated toxicity in mouse preimplantation embryos.

As shown in Figure 5E, real time qRT-PCR analysis showed that rapamycin pretreatment decreased mTOR, Cyclin D1, and c-Myc expression levels. This effect was accompanied by decreased expression levels of G_1_-associated proteins, such as CCDN1 (Cyclin D1) and c-MYC. Considering blastocyst with developmental defects by CSNPs treatment exhibited a delay in mitochondrial DNA (mtDNA) replication, we analyzed ICM-, TE-, and three germ layer-specific marker gene expressions, which involved in development of preimplantation embryos, using the RT^2^ Profiler PCR Array (Figure 5F). We found that there were substantial differences among the CSNPs-treated, control, and rapamycin+CSNPs-treated groups. Surprisingly, differentially expressed genes, namely, Lif, Stat3, Klf4, Sox2, Cdx2, Emos, Krt8, B3gnt, Gata6, Bmp4, and Fgf5, were all substantially down regulated upon exposure to CSNPs, whereas most of the down regulated genes were rescued by rapamycin treatment. Overall, these results suggest that rapamycin pretreatment may promote the transition from the morula stage to the blastocyst stage, and blastocoel formation, thus preventing cell cycle arrest and apoptosis induced by CSNPs treatment.

### CSNPs treatment alters epigenetic reprogramming

**Figure 6:**
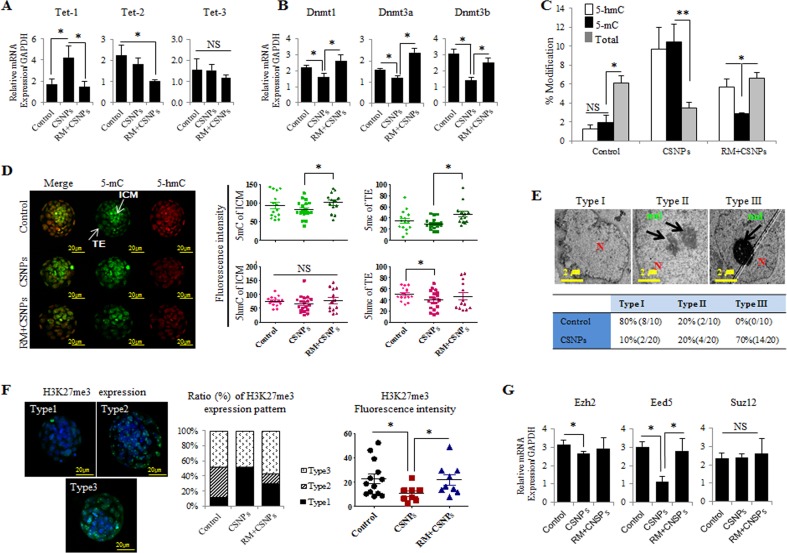
CSNPs-altered epigenetics in preimplantation embryos **A**. Real time qRT-PCR analysis of the Tet family (Tet-1, Tet-2, Tet-3) and **B**. the Dnmt family (Dnmt1, Dnmt3a, Dnmt3b). **C**. 5-mC to 5-hmC analysis. **D**. Immunostaining of 5-mC and 5-hmC. The fluorescence intensities of 5-mC and 5-hmC were measured in both the ICM and the TE. **E**. Heterochromatin condensation was observed in the control and CNSPs-treated groups using TEM. To calculate each type of heterochromatin and based on the condensation, we divided the embryos into 3 groups: Types I, II and III. Transcriptional silencing is associated with the targeting of genomic sequences to repressive (heterochromatic) nuclear compartments. **F**. The H3K27me3 expression level was analyzed using immunofluorescence staining. Embryos were divided into Types I, II, and III by their expression patterns. **G**. The relative expression levels of methylase (Ezh2) and its co-factors (Eed5, Suz12) were measured using real time qRT-PCR analysis. *, **, and NS indicate *p*<0.05, *p*<0.01, and not significant, respectively. RM indicates rapamycin.

Epigenetic mechanisms are essential elements for the regulation of cellular differentiation and the maintenance of cell type-specific gene expression patterns [45]. Ten-eleven translocation proteins (TETs) enzymes (TET-1/−2/−3) convert 5-methylcytosine (5-mC) to 5-hydroxymethylcytosine (5-hmC) in various embryonic and adult tissues [50, 51]. To further characterize how CSNPs treatment or rapamycin pretreatment modulates the epigenetic reprogramming of preimplantation embryos, we measured the mRNA expression levels of Tets, DNA methyltransferase (Dnmts), density of 5-mC, and 5-hmC signals. Even though the Tet-2 and -3 mRNAs expression in CSNPs-treated group do not show a statistical difference compared to the control group, Tet-1 mRNA in the CSNPs treated groups was more strongly expressed (Figure 6A). Of note, Tet-2 mRNAs expression levels were significantly lower in the blastocysts pretreated with rapamycin compared to those treated with CSNPs alone. There was no significant difference in the expression of Tet-3 mRNA among the three groups.

DNA methylation is associated with repressive, inactive chromatin and is a key stable and heritable epigenetic modification [52]. The majority of 5-mC occurs at CpG dinucleotides that are modified up to 70-80% in a cell type specific manner in human cells and are faithfully transmitted to daughter cells during cell division [53, 54]. DNA methylation is catalyzed by DNMT1, DNMT3a and DNMT3b. DNMT1 maintains existing DNA methylation patterns, whereas DNMT3a and DNMT3b are mainly involved in the de novo establishment of DNA methylation marks [55, 56]. We examined whether CSNPs-mediated DNA demethylation may be a DNMTs-mediated process and also to examine whether CSNPs is directly targeting of DNA methylation through the DNMTs. To determine the impact of CSNPs on the expression of Dnmt families, the embryos were treated with CSNPs, and their relative mRNA expression levels were measured. Interestingly, CSNPs-treated groups exhibited significant reductions in Dnmt1, Dnmt3a, and Dnmt3b mRNAs expression. Conversely, rapamycin pretreatment rescued the effect caused by CSNPs, in which the relative Dnmt1, Dnmt3a, and Dnmt3b mRNAs expression levels were comparable with the control (Figure 6B). 5-hmC is known to play an important key intermediate for active DNA demethylation pathway during DNA repair [57]. The widely used bisulfite sequencing technique does not distinguish between 5-mC and 5-hmC. Therefore, in this study we used synthetic oligonucleotides with different distributions of cytosine as templates for generation of DNAs containing 5-mC and 5-hmC. The resulting CSNPs treated embryos shows higher level of 5-mC and 5-hmC than untreated groups. However, the embryos treated with rapamycin+CSNPs exhibited lower level of 5-mC and 5-hmC than CSNP treatment only. It suggests that CSNPs play crucial role in methylation process of embryonic cells (Figure 6C).

Next, we examined the density of 5-hmC and 5-mC signals from fully developed to arrest embryos (Figure 6D and Figure S4A). In control-derived blastocyst stage embryos, 5-mC signal intensity in ICM cells was stronger than those of TE cells, whereas 5-hmC signals in ICM cells was weaker than those of TE cells. After rapamycin pretreatment, 5-mC and 5-hmC signal levels were increased in ICM and TE cells. This finding suggests that rapamycin recovered the CSNPs-induced epigenetic changes. In 100% blastocoels treated with CSNPs, the signal levels of 5-hmC were slightly decreased in the ICM and TE, whereas on rapamycin+CSNPs group, intensity of the 5-mC signal in the TE and ICM cells resulted in a dramatic increase compared to CSNPs group (Figure 6D). In 80% blastocoels treated with CSNPs, the expression levels of 5-mC and 5-hmC signals were significantly reduced when compared to control (Figure S4B) and surprisingly rapamycin+CSNPs treated 80% blastocoels shows further reduced level of both 5-mC and 5-hmC signals (Figure S4B). In 30% blastocoels treated with CSNPs or rapamycin+CSNPs, the expression levels of 5-mC and 5-hmC were not significantly different between CSNPs and rapamycin+CSNPs treated groups (Figure S4B). However, the expression levels of 5-mC and 5-hmC signals were significantly different from control on delayed embryonic development. Taken together, this finding indicates that CSNPs may regulate active or passive DNA methylation in the TE cells of mouse blastocysts.

Eukaryotic interphase nuclei are highly compartmentalized structures and have two types of chromatin, such as euchromatin and heterochromatin, which are potentially active and inactive, respectively [58]. The architectural organization of the nucleus and the regulation of transcription are functionally linked. There is also significant evidence that shows that transcriptional silencing is associated with the targeting of genomic sequences to repressive (heterochromatic) nuclear compartments. Furthermore, it has long been known that the methylation of particular DNA sequences is often linked to their transcriptional inactivity [59]. Next, we wanted to observe the effect of rapamycin on the chromatin display using TEM. The TEM results were used to determine the ratio of heterochromatin to euchromatin, the location of the nucleus, and its shape. Each cell showed three types of chromatin. Interestingly, control group showed a slight condensed chromatin, whereas the CSNPs-treated groups showed more condensed chromatin than the control group (Figure 6E and Figure S3).

In this study, we examined H3K27me3 localization and expression and found that there were significant changes in the modifications of both the ICM and TE. Depend on blastocyst development stages, we divided into 3 groups. Of note, CSNPs treated group shown type I and III expression patterns, but not for type II. The intensity of the H3K27me3 level was lower in CSNPs-treated blastocysts compared to control blastocysts; however, rapamycin rescued the side effects caused by CSNPs treatment (Figure 6F). Finally, we were also interested in studying the effects of CSNPs on the expression of the methyltransferase of H3K27me3 and PRC2 complex such as the Ezh2 co-factors, Eed5 and Suz12. We found that global levels of H3K27me3 decreased in the CSNPs-treated groups, corresponding to the time of major embryonic genome activation. Ezh2 and Eed5 mRNA expression was remarkably decreased in the CSNPs-treated embryos compared to the control; conversely, Suz12 expression was not significantly different from the control (Figure 6G). Interestingly, pretreatment with rapamycin rescued the side effects caused by CSNPs in the embryos in all cases. Previously, a study from bovine oocytes and pre-implantation embryos examined the expression levels of H3K27me3, Ezh2 and its co-factors, Eed5 and Suz12 genes [60]. The global level of H3K27me3 was highest in oocytes, decreased during initial cleavages, and increased again from the 8-cell to the blastocyst stage [61]. Taken together, CSNPs treatment alters the epigenetic regulatory mechanism of genome-wide gene expression.

### Rapamycin rescues suppressive effects of CSNPs on postimplantation embryo development

**Figure 7:**
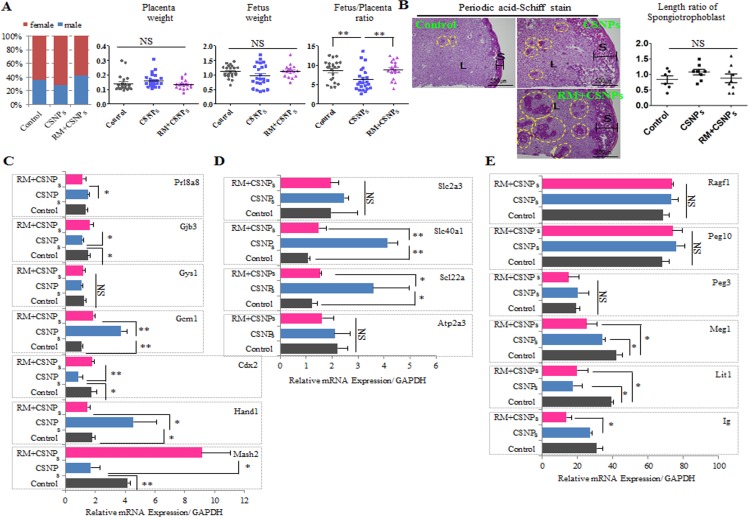
Comparison of sex ratio, placenta weight, and fetus weight in control and CSNPs or rapamycin+CSNPs treated groups Mouse blastocysts were treated with or without CSNPs, and with rapamycin+CSNPs, and transferred into uteri of pseudopregnant mice. Pups were recovered by caesarian section at gestational day 20. **A.** The sex ratio, placenta weight, fetus weight, the fetus and placenta ratio were analyzed. The rapamycin+ CSNPs-treated group showed a fetus/placenta ratio comparable with the control and the CSNPs-treated groups. **B.** Morphology analysis of placenta by PAS staining from the control, the CSNPs, and CSNPs+rapamycin treated group. PAS positive staining was observed in the decidua and junctional layers. In the spongiotrophoblasts, deep purple color indicates glycogen cells. Of note, CSNPs-treated placenta exhibited a slightly high glycogen cell number/length. CSNPs or CSNPs+rapamycin treated groups showed a lot of mislocation of spongiotrophoblasts. **C.** The relative expression levels of critical developmental genes, including Mash2, Cdx2, Hand1, Gys1, Gcm1, Prl8a8, and Gjb3, in the E20 placenta. **D.** Rapamycin rescued abnormal regulation of placental nutrient transporters caused by CSNPs treatment. **E.** Comparison of Ig, Lit1, Meg1, Peg3, Peg10, and Ragf1 mRNAs expression in full-term derived placentas. *, **, and NS indicate *p* < 0.05, *p* < 0.01, and not significant, respectively. RM indicates rapamycin.

To determine the effects of rapamycin on CSNPs-induced developmental defects, blastocysts derived from the control group, the group treated with CSNPs only, and the group treated with rapamycin+CSNPs were transferred into recipients, respectively and the numbers of blastocysts with implantation bleeding (or spotting) were calculated in the uteri of recipient mice. The implantation ratio of the CSNPs-treated group was significantly lower than the control or both of rapamycin+CSNPs-treated group. The CSNPs definitely had a significant negative effect on development, and rapamycin treatment showed a rescue effect on postimplantation development (Figure S6A). As shown in Figure 7A, the sex ratios of offspring were determined in all treated groups, including the control group. The CSNPs-treated groups had smaller male ratios compared to both the control and the rapamycin- and CSNPs-treated groups. The CSNPs-treated groups showed higher placental weight and lower fetus weight, whereas the rapamycin- and CSNPs-treated group showed the reverse order (Figure 7A). These findings indicate that CSNPs exposure at the morula stage for 24 h reduces embryo implantation, whereas rapamycin treatment has a potential rescue effect on CSNPs-induced postimplantation development (Figure S6B).

Normal placental development is important for embryonic growth, and placental dysfunction leads to miscarriage and fetal growth restrictions [10, 62, 63]. In mice, there are three distinctive trophoblastic cell structures in developing placenta that are formed by embryonic day 10 and persist throughout gestation: an inner labyrinthine layer, an intermediate spongiotrophoblast layer, and an outermost layer of trophoblast giant cells [64, 65]. To investigate the role of CSNPs treatment in placental dysfunction, we examined placental abnormalities through the observation of the spongiotrophoblast layer using Periodic Acid-Schiff (PAS) staining. There was a marked increase in the spongiotrophoblast layer in CSNPs-derived placentae, and we found many mislocated spongiotrophoblast cells in CSNPs treatment, and rapamycin+CSNPs treated groups (Figure 7B and Figure S6C). Of note, spongiotrophoblast areas in the cells in some of the CSNPs-treated embryo derived placentae were slightly increased, but most increases were not significant. However, the length and area of the spongiotrophoblasts in the CSNPs-treated group were different compared to the control group, but rapamycin treatment rescued the defects in spongiotrophoblast length and area (Figure 7B). Defective placenta may affect the normal branching and/or survival of the embryonic labyrinthine vascular system. Abnormalities of the labyrinthine layer can be raised as a consequence of abnormalities in the allantoic mesenchyme or the trophoblast [65]. An intact spongiotrophoblast layer is required for normal development of the labyrinthine trophoblast layer [66]. Taken together, rapamycin might be used to rescue placental abnormalities caused by CSNPs.

Next, we investigated the rescue effect of rapamycin on the expression profiles of various genes involved in placental development. Among these genes, the CSNPs-treated groups showed downregulation of Msh2, Cdx2, Gjb3, and Gbe1 mRNAs expression levels, whereas Hand1 and Gcm1 mRNAs expression levels were upregulated; Gys1 and Prl8a8 mRNAs expression showed no significant difference between the treated and untreated groups (Figure 7C). Interestingly, regardless of whether the genes were upregulated or downregulated in the CSNPs-treated conditions, all of these genes were rescued by rapamycin pretreatment.

Finally, we analyzed nutrient transport gene expressions, such as Atp2a3, solute carrier family 22 member 3 (Slc22a3), and solute carrier family 40 (iron-regulated transporter), member 1 (Slc40a1) (Figure 7D). These genes were expressed at higher levels in the presence of CSNPs, whereas rapamycin treatment modulated their expression levels so that they were comparable to those of the control. Also, we evaluated the expression of imprinted genes involved in the methylation process (Figure 7E). Of these, the expression levels of Lit1 and Meg1 mRNAs in the CSNPs-treated group were significantly downregulated compared with those of the control. However, rapamycin did not show any significant rescue effect on the CSNPs-induced downregulation. Conversely, the expression levels of Peg3, Peg10, and Ragf1 mRNAs were slightly upregulated in the presence of CSNPs, and in the group treated with rapamycin, rapamycin rescued the effect caused by CSNPs. From our analysis, we conclude that rapamycin is essential to the rescue of placental development defects caused by CSNPs and that CSNPs have a negative effect on normal homeostasis in mouse embryos.

### Temporal toxic effects of CSNPs on primordial follicles, developing follicles, granulosa, and theca cells by direct injection of CSNPs into tail vein of female mice

**Figure 8:**
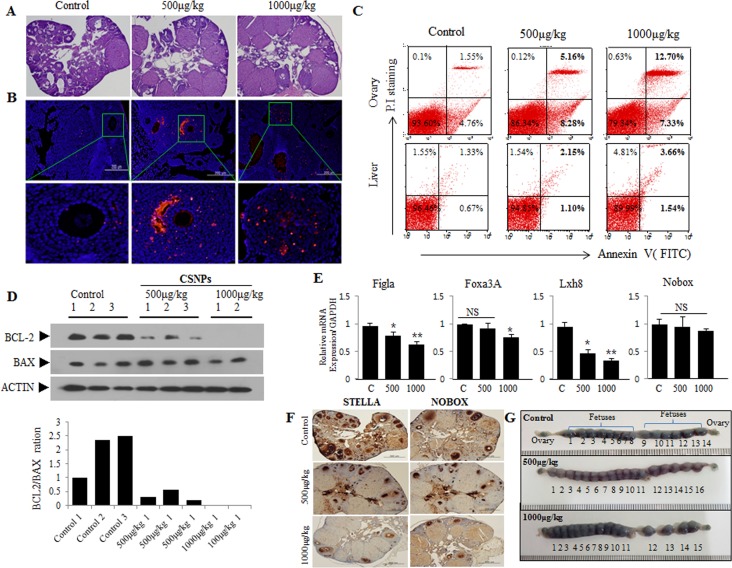
Short-term toxic effects on ovary, primodial follicles, and developing fetuses after injection of CSNPs into vein of female mouse Two different doses (500 or 1000 μg/kg per body weight) of CSNPs were injected into tail vein of female mice. Ten days later, ovaries were recovered from CSNPs-injected female mice. **A.**-**D.** shows HE staining, TUNEL assay, Flow cytometry, Western blot analysis, respectively. In CSNPs-injected groups, a lot of apoptosis (red color) was observed. BCL-2 expressions in CSNPs-injected groups are significantly decreased or undetectable. **E.**-**F.** Real time qRT-PCR and immunohistochemistry analysis. Expression of Fig 1a, Foxa3A, Lhx8, and Nobox mRNAs, which is a biomaker of primordial follicle, were compared in control and CSNPs-treated groups. **G.** Comparison of developing fetusus between control and CSNPs treated groups. At 10 day after injection of CSNPs into vein of female mice, female mice were mated with fertile male mice and confirmed varginal plug at next morning (gestational day 0.5). Uteri from each female groups were recovered at gestational day 9.5 (at 19.5 days after CSNPs injection), stained with Chicago blue dye, and counted developing fetuses.

To corroborate the *in vitro* data, we examined the potential adverse effects of CSNPs *in vivo*. Female mice were intravenously injected at concentration of 500μg CSNPs/kg or 1000μg CSNPs/kg body weight, and 10 days later ovaries were subjected to examination. The results from *in vivo* studies suggest that CSNPs-treated groups showed significant reduction in the number of developing follicles, compared to control group (Figure 8A). Furthermore, TUNEL assay also showed significant number of TUNEL positive granular and theca cells (Figure 8B), whereas the number of TUNEL positive cells rarely observed in control group. Therefore, ovarian steroidogenesis, which plays a pivotal role for follicle development, is expected to be hampered because of CSNPs-induced loss of granular and theca cells. Further, to substantiate apoptotic property of CSNPs, we performed flow cytometry analysis (Figure 8C). The result from flow cytometry analysis suggest that CSNPs enhances the level of late apoptosis up to 3.3 folds for 500μg of CSNPs/kg and 8.19 folds for 1000μg of CSNPs/kg treated mice compared to control group which showed up to 1.55% late apoptotic cells. However, the observation indicated that CSNPs-treated mice showed only moderate apoptosis in liver compared to ovaries. To support the data obtained from *in vivo*, we performed western blotting analysis which suggest that the expression of BCL-2 protein were significantly decreased at lower dose of CSNPs (500μg/kg) and not detectable at higher dose (1000μg/kg) (Figure 8D). All these findings indicated that CSNPs injection causes adverse effects on ovarian function.

Real time qRT-PCR (Figure 8E) and immunohistochemistry (Figure 8F) analysis was performed using primordial follicle biomarkers, STELLA and NOBOX. The result showed that primordial follicles are significantly reduced at 10 days after CSNPs treatment. Further, to examine *in vivo* toxicity of the newly ovulated oocytes and fertilized embryos, we recovered blastocyst stage embryos from gestational day 3.0 mice (12.5 day after CSNPs injection). Notably, CSNPs-injected mice produced a lot of blastocyst embryos (500μ/kg *vs*. 1000μ/kg: 39±6.4 *vs*. 26.1±9.3 per mouse) than those (22±9 per mouse) of control mice. However, there is no significant difference between treated and control group in total cells, CDX2 and OCT3/4-positive cells (Figure S5). Of note, CSNPs treated pregnant mice have equal or more number of fetuses than those of control group, indicating that newly ovulated oocytes around 10 days after injection of CSNPs into vein of female mice have no effect (Figure 8G). Taken together, our observations indicated that CSNPs injection into female mice required long term exposure rather than short-term to impart its fetotoxic effect.

## CONCLUSIONS

The increased use of nanomaterials in our daily life has raised concerns regarding their potential risks to human health and their effects on biological behaviors at the cellular, subcellular, and protein levels and in terms of developmental delays. In particular, the use of CSNPs in nanomedicine is ever increasing, and it is important to understand their targeted and non-targeted effects. Here, we report a comprehensive collection of the significant nanoreprotoxicity of CSNPs in blastocysts through their cellular uptake and their effects on various signaling cascades, such as oxidative stress, ER stress, and autophagy. Furthermore, we have demonstrated the rescue effects of rapamycin in the amelioration of CSNPs-induced detrimental effects in blastocysts. With all of these data, we summarized the mechanisms of the effects of CSNPs on preimplantation embryos (Figure S7). CSNPs-treated embryos have detrimental effects in their placentae that are linked to their structural and functional aspects. Additionally, we analyzed the epigenetic regulatory mechanisms of CSNPs-treated embryos by immunofluorescence staining. Combined with advanced microfabrication techniques, our studies indicated that the CSNPs delivery system could be applied to other multiple cells, including germ cells, with higher safety. This study also provides a significant contribution to future research, which should focus on the exact mechanisms of the effects of CSNPs and should concentrate on producing biocompatible and safer nanomaterials.

## MATERIALS AND METHODS

### Synthesis and characterization of CSNPs

CSNPs were prepared as described earlier [18]. Briefly, chitosan solutions were prepared by dissolving purified and sonicated chitosan in a 0.1M acetic acid solution until the solution was transparent. Tripolyphosphate (TPP) was dissolved in deionized water at a concentration of 1 mg/ml. The chitosan solution was mixed with the TPP solution, and the formation of chitosan-TPP NPs began spontaneously *via* the TPP-initiated ionic gelation mechanism. The size of the synthesized particles was measured using a Zetasizer Nano ZS90 (Malvern Instruments Ltd, Worcestershire, UK). Furthermore, the shape and morphology was determined using TEM (JEM-1200EX). Ten microliters of the nanoparticle suspension was placed on a copper grid, and the excess liquid was removed with a piece of filter paper. The grid was then air-dried.

### Animal

The mice were housed in wire cages at 22±1°C under a 12 h light-dark cycle with 70% humidity and had free access to water and food. All experiments were conducted in accordance with the Konkuk University Guide for the Care and Use of Laboratory Animals (IACUC approval number: KU12044).

### Treatment

The dose of CSNPs was optimized as 100μg/ml based on previous study [18]. For *in vivo* study, we used two different doses such as 500μgof CSNPs/kg and 1000μg of CSNPs/kg. Two different doses, 500μg of CSNPs/kg or1000μg of CSNPs/kg, are injected into tail vein of female mouse for *in vivo* study.

### Collection of mouse morula-stage embryos

The collection of morula-stage embryos from mice was performed as described earlier [18]. Female ICR mice (age 6-8 weeks) were superovulated by the injection of 5IU of the pregnant mare's serum gonadotropin (PMSG), followed 48 h later by an injection of 5IU of human chorionic gonadotropin (hCG) and were then mated with fertile male ICR mice. The day a vaginal plug was found was defined as day 0.5 of gestation. Morulae stage embryos from plug-positive females mice were obtained by flushing the uterine tubes at 2.5 days post-mating using CZB-HEPES medium. After the morula-stage embryos were collected, they were cultured with KSOM [67] including CSNPs or rapamycin+CSNPs for 24-28 h at 37°C at 5% CO_2_. All reagents were purchased from Sigma-Aldrich (St. Louis, MO, USA).

### Localization of CSNPs in embryos by transmission electron microscopy (TEM)

After fixation of arterial samples in 2.5% glutaraldehyde (TED PELLA, CA, USA) in PBS (pH 7.2), specimens were post-fixed in 1% osmium tetroxide (Heraeus, Hanau, Germany), dehydrated in graded ethanol and propylene oxide (Acros Organics, USA), and then embedded in Epoxy resin (mix with Nadic Methyl Anhydride (NMA) and Dodecenyl Succinic Anhydride (DDSA) and DMP-30, all reagents from Polysciences (PA, USA). Serial ultrathin sections were cut using an LKB-III ultratome (LEICA, Wetzlar, Germany). Ultrathin sections were stained with uranyl acetate (TED PELLA, CA, USA) and lead citrate (TED PELLA, CA, USA) and were examined with the aid of a Hitachi H7600 electron microscope (Hitachi, Japan) at an accelerating voltage of 100 kV.

### Mitochondrial analysis

A Rhod2-AM (Molecular Probes, Eugene, OR) probe was used to determine the mitochondrial Ca^2+^ level [68]. Rhod2-AM possesses a net positive charge, which facilitates its sequestration into the mitochondria because of its membrane potential-driven uptake. The use of Rhod2-AM enhances the selectivity for mitochondrial loading because this dye exhibits Ca^2+^-dependent fluorescence only after it is oxidized, which occurs preferentially within the mitochondria. Cells were treated with CSNPs and incubated for 12h, and the cells were then harvested, washed, and suspended in PBS containing Rhod2-AM (1μM). For image analysis, the cells were loaded with Rhod2-AM and incubated for 30 min at 37°C. Cells were washed, and the stained cells were mounted onto a microscope slide with mounting medium (Vector Laboratories, USA). For mitochondrial staining, embryos in culture were incubated in 160nM MitoTracker (Molecular Probes, OR, USA) in DMEM before being fixed and immunostained. For green fluorescent protein (GFP) visualization, cells were only fixed and washed. Microscopic images were collected using an Olympus BX-UCB microscope and were processed using DP controller software. Quantitative analysis of fluorescence intensity was performed using MeTaMorph image analysis software (Molecular Devices, California, USA).

### ER analysis

Image analysis for ER staining was achieved by seeding cells on a coverslip-loaded six-well plate at 50 blastocysts/ml. At 16 h after plating, blastocyst were treated with CSNPs. Twelve hours later, the ER-Tracker Blue-White DPX (Molecular Probes, Eugene, OR) probe was added to blastocyst and was incubated for 30 min under the same growth conditions. The loading solution was removed, and blastocysts were then washed with PBS. Microscopic images were collected using an Olympus BX-UCB microscope and processed using DP controller software. The quantitative analysis of fluorescent intensity was performed using MeTaMorph image analysis software (Molecular Devices, California, USA).

### Western blotting analysis

Approximately 30 blastocyst or ovary cells was lysed in RIPA (GenDOPET, Texas, USA) buffer containing protease inhibitors, and then subjected to 10% SDS-PAGE. The separated proteins were transferred to a PVDF, which was then pretreated with 5% skim milk for blocking and incubated with primary antibodies as follows: GRP78 (Abcam, UK) and LC3 (Cell Signaling, Boston, USA) for blastocysts samples, BAX (Abcam, UK) and BCL-2 (Santa Cruz Biotechnology, TA, USA) for ovary samples. Blots were then incubated with anti-rabbit or anti-mouse IgG antibody conjugated to horseradish peroxidase. The protein bands were visualized using an ECL plus Western Blotting Detection System (Amersham Pharmacia Biotech, USA). The membrane was then washed and reblotted with actin antibody for an internal control. Densitometric quantification was performed with ImageJ software (NIH).

### Detection of intracellular ROS

To detect intercellular ROS in living embryos, we used H2DCF-DA from Sigma-Aldrich (St. Louis, MO, USA). H2DCF-DA was prepared in DMSO immediately prior to loading. Embryos were incubated with 10μM H2DCF-DA for 10 minutes and observed under afluorescence microscope (Olympus, Japan), with an excitation wavelength of 480 nm and an emission wavelength of 505-530 nm.

### mRNA extraction and amplification

Total RNAs was extracted from blastocysts using Dynabeads mRNA Direct Kit (ThermoFisher, USA) according to the manufacturer's instructions. Real time qRT-PCR was conducted using a Vill7 (Applied Biosystems, OR, USA) and SYBR Green as the double-stranded DNA-specific fluorescent dye (Applied Biosystems, OR, USA). Target gene expression levels were normalized to *GAPDH* gene expression, which was unaffected by CSNPs treatment. The mitochondrial DNA (mtDNA) content was determined by comparing the ratio of mtDNA to nuclear DNA, as measured by real time qRT-PCR. The real time qRT-PCR primer sets are shown in Tables S2-4. Real time qRT-PCR was performed independently in triplicate for each of the different samples, and the data are presented as the mean values of the gene expression levels measured in the CSNPs-treated samples *versus* the controls.

### RT2 profiler PCR array

Changes in gene expression were analyzed using a pathway-focused Mouse Embryonic Stem Cells PCR array (SABiosciences, Valencia, CA, USA). Data were analyzed using SABiosciences RT^2^ Profiler PCR Data Analysis software, available at http://pcrdataanalysis.sabiosciences.com/pcr/arrayanalysis.php, and were considered significant at >1.5-fold change. Relative quantitation of each gene was performed by normalization to four housekeeping genes (Actb, B2m, Gapdh, and Gusb).

### Terminal deoxynucleotidyl transferase dUTP nick-end labeling (TUNEL) assay

Apoptosis was assessed using the In Situ Cell Death Detection Kit (Roche, West Sussex, UK) and 4',6-diamidino-2-phenylindole (DAPI) dual staining (Vector Laboratories, CA, USA). Briefly, the cells and ovary tissues in each group were prepared, washed twice with D-PBS and then incubated with TUNEL reaction mixture for 60 min at 37°C. The cells undergoing apoptosis were detected using an Olympus BX-UCB microscope and were processed using DP controller software. At least 20 blastocysts were analyzed for each determination.

### Immunostaining and confocal imaging

Embryos were fixed in 4% paraformaldehyde for 20 min and permeabilized for 20 min with PBS containing 0.2% Triton X-100. Permeabilized embryos were blocked overnight at 4°C in 1% bovine serum albumin (BSA) and 0.1% Triton X-100 in PBS before incubation with the primary antibodies anti-5-mC (Eurogentec, Seraing, Belgium), anti-5-hmC (Active Motif, CA, USA), LC3 (Novus, CO, USA), OCT3/4 (Santa Cruz Biotechnology, TA, USA), H3K27me3 (Millipore, MA, USA), and CDX2 (Biogenex, CA, USA) for 2 h in a humidified chamber at room temperature. For 5-mC and 5-hmC immune stains, samples were prepared as described by Dean group [69]. Embryos were preincubated with 4N HCl for 10 min at room temperature and then with 100 mM Tris-HCl for 20 min after the blocking step. The embryos were washed several times in 0.05% Tween 20 in PBS (PBST), transferred to a secondary antibody mixture of Alexa Fluor 568 goat anti-mouse and Alexa Fluor 488 goat anti-rabbit (Molecular Probes, USA), and incubated at room temperature for 30 min. Confocal images were acquired using an Olympus Fv1000 microscope and were processed using FV10-ASW 2.0 Viewer. Fluorescent images were acquired using an Olympus BX-UCB microscope and were processed using DP controller software. The quantitative analysis of the fluorescent intensity was performed using MeTaMorph image analysis software (Molecular Devices, California, USA).

### Immunohistochemistry (IHC)

At 10 days after CSNPs injection *via* tail vein, ovary was fixed with 4% paraformaldehyde and embedded in paraffin. Sections were stained with Hematoxylin for light microscopy. After deparaffination and rehydration in PBS, sections were blocked in background Sniper solution. After washing, the samples were incubated with specific primary antibodies for STELLA (MilliporeCompany, MA, USA) and NOBOX (Cell Signaling, Boston, USA). Samples were then stained with ImmPACT^TM^ DAB peroxidase substrate (Vector Laboratories; CA, USA) to visualize the signal and observed using fluorescence microscopy (Olympus, Japan).

### Embryo transfer

Female mice were mated with vasectomized male mice to induce recipient mice. After mating, the females were checked for the presence of a vaginal plug, and upon finding it, they were assumed to be on day 0.5 of pseudopregnancy. Blastocysts were transferred through the utero-tubal junction of the uterus of surrogate mother under inhalation anesthesia with isoflurane (Isoba Vet Schering-Plough, New Jersey, USA). Blastocysts were transferred through the utero-tubal junction into the uterus of surrogate mother as synchronous between the embryo and maternal environment.

### Analysis of implantation

Control or CSNPs treated females were mated with wild type fertile males to induce pregnancy. Implantation sites were examined at gestational day 9.5 (19.5 days after CSNPs injection) and visualized by intravenous injection of a Chicago blue dye solution (0.1ml of 1% Chicago blue).

### Flow cytometry analysis

Ovary were recovered from CSNPs injected female mice, minced, and trypsinized to make single cells. Single cells are washed in PBS, centrifuged at 1200g, and resuspened in binding buffer. TUNEL analysis was performed to measure the degree of cellular apoptosis using an *EzWay Annexin V-FITC apoptosis Detection kit* (Komabiotech, Seoul, Korea) according to the manufacturer's instructions. Cells were characterized using a FACSCalibur cell analyzer and the data were analyzed using CellQuest software (BD Biosciences, New Jersey, USA). For each runs, 10^4^events were collected. We counted at least 10,000 cells/tube for flow cytometry analysis in each experiment, respectively.

### Periodic acid-schiff (PAS) staining

Fixed placental discs were bisected through the attachment of the umbilical cord and then embedded in paraffin wax. At least five placentae (from different litters) in each group were examined. Five-micrometer cross-sections were obtained and stained with hematoxylin and PAS-hematoxylin (Sigma-Aldrich, St. Louis, MO, USA). The border between the labyrinth and spongiotrophoblasts was identified visually. The total cross-sectional areas of labyrinthine zones were measured and calculated using ImageJ software (National Institutes of Health, Bethesda, MD).

### DNA glycosylation, digestion and real time qRT-PCR

To analyze and quantitate 5-mC and 5-hmC within a specific locus, we used an EpiMark 5-hmC and 5-mC Analysis Kit (NEB, MA, USA) according to the manufacturer's instructions. In brief, DNA was isolated from embryos at the blastocyst stage (*n* = 10). DNA was then subjected to T4 Phage β-glucosyltransferase (T4-BGT, NEB) treatment for 18 h. Glycosylated DNA was digested with 40U of HpaII, 100U of MspI or no enzyme (mock digestion) at 37°C for 18 h, which was followed by treatment with Proteinase K (PK) for 30 min at 40°C. The subsequent inactivation of PK was performed at 98°C for 10 min. HpaII- and MspI-resistant fractions were used for real time qRT-PCR with primers that were designed around the MspI (HpaII) site. DNA subjected to the glycosylation treatment that was not completely digested by MspI at the MspI site was considered to contain 5-hmC at this site. The real time qRT-PCR reaction (25 μl) consisted of 2μl of DNA, 12.5μl of SYBR Green master mix (TaKaRa, Japan), 9.5μl of RNase-free water and 0.5μl of both forward and reverse primers (10 pmol) for each gene. The real time qRT-PCR protocol consisted of a denaturing cycle of 10 min at 95°C and 40 cycles of PCR (95°C for 10s, 60°C for 30 sec, 72°C 20 sec). The relative amounts of DNA were analyzed using the 2^−ΔΔCT^ method.

### Data analysis

All experimental data are presented as the mean± SD. Each experiment was performed at least three times and was subjected to statistical analysis. The results from the three representative experiments are presented in each figure. For statistical analysis, one-way analysis of variance (ANOVA) was first performed to determine whether there were differences among groups (*P* < 0.05). Fisher's post-test was then performed to determine the significant differences between pairs. A value of *P* < 0.05 was considered significant. Statistical tests were performed using StatView software version 5.0 (SAS Institute Inc., Cary, NC).

## SUPPLEMENTARY MATERIALS TABLES AND FIGURES


