# Supplementary material for: Rapamycin ameliorates chitosan nanoparticle-induced developmental defects of preimplantation embryos in mice
Source: Oncotarget. 2016 Jul 24;7(46):74658–77. doi: 10.18632/oncotarget.10813 (PMC5342693; doi:10.18632/oncotarget.10813)
Supplement: Supplementary file 1 [file oncotarget-07-74658-s001.pdf]

Rapamycin ameliorates chitosan nanoparticle-induced developmental defects of preimplantation embryos in mice

Supplementary Material

Supplementary Table 1: Effect of in vitro development in chitosan nanoparticles treated mouse embryo

Treatment	No. of morula	100% BL	80% BL	30% BL	0% Mo.
Control	59	48 (80.9±1.4[a])	8 (14.4±2.1[b])	2 (3.0±1.8[c])	1 (1.7±1.7[b])
CSNPs	66	29 (45.2±5.8[c])	20 (28.2±5.3[a])	9 (13.5±2.3[a])	7 (10.9±4.3[a])
Rapamycin 5 + CSNPs	71	40 (58.8±4.9[b])	20 (26.0±4.6[a])	7 (9,8±2.3[b])	4 (5.4±1.9[ab])
Rapamycin 10 + CSNPs	87	52 (60.5±2.6[b])	24 (27.1±2.5[a])	7 (7.6±3.1[b])	4 (4.8±1.7[ab])
Rapamycin 50 + CSNPs	82	47 (57.4±3.6[b])	22 (26.7±2.5[a])	8 (9.9±1.9[b])	5 (6.0±0.7[ab])
Rapamycin 100 + CSNPs	79	47 (60.3±2.9[b])	18 (21.6±3.7[ab])	9 (11.8±1.7[ab])	5 (6.3±0.8[ab])

Supplementary Table 2: Primers used for quantitative real-time polymerase chain reaction

Gene		Primer	Gene		Primer
Atg4a	F	CCCTCACACAACCCAGACTT	Ip3R type1	F	CTAACCCCTCTCCTGGTCCT
	R	CCCCTGTGGTTGTCACTTCT		R	CACCAAAGCCACCAAGAACT
Atg4b	F	CCACAAGGTAGCAAGAGA	Junk1	F	GCCACAAAATCCTCTTTCCA
	R	GAAGTCAAGGGACAAGATATG		R	CACATCGGGGAACAGTTTCT
Atg5	F	GGAGAGAAGAGGAGCCAGGT	LC3	F	TTCTTCCTCCTGGTGAATGG
	R	TGTTGCCTCCACTGAACTTG		R	GTGGGTGCCTACGTTCTCAT
Atg6	F	GTCTTATGAGATGAGGAATG	mTOR	F	ACACAGTAATCCTTCAGA
	R	AATGGCAGGATACTTCTT		R	GCAATGTTTATGATGAGTTT
Atg7	F	CAGTAGCCTGTAGAATAAC	Perk	F	GGGAAAACGGTTCTGAGACA
	R	GTAGGTGTGTCTGTAATC		R	GCTGACCAGCTAGTCTTGGG
Atf6	F	AACCAGTCCTTGCTGTTGCT	Sirtuin-1	F	AAGGAGCAGATTAGTAAGC
	R	CTTCTTCTTGCGGGACTGAC		R	GCACCGTGGAATATGTAA
Bax	F	CGAGCTGATCAGAACCATCA	Sirtuin-3	F	TACAGGCCCAATGTCACTCA
	R	GAAAAATGCCTTTCCCCTTC		R	ACAGACCGTGCATGTAGCTG
Bcl2	F	TAAGCTGTCACAGAGGGGCT	Sirtuin-6	F	CTGGTCTGGAACTCACTGCT

	R	TGAAGAGTTCCTCCACCACC		R	CGGGTGTGATTGGTAGAGAG
Beclin1	F	ACAGTTCTATAACAAGTGA	Tet-1	F	GCCTGTTTCATTTCCGTTGT
	R	GGGTTCTTTGCTATACAT		R	GTCCCTGACGTAGGACCAAA
Calpain2	F	CACCCTCACCTGTGACTCCTAT AA	Tet-2	F	GTTCTCAACGAGCAGGAAGG
	R	ATCCTCCTCATCTTCGTCTT		R	TGAGATGCGGTACTCTGCAC
Caspase 33	F	AGGGGTCATTTATGGGACA	Tet-3	F	GGTCACGCATCGTCCTTATT
	R	TACACGGGATCTGTTTCTTTG		R	TTTTAGGATGGGCGTGTTTC
Chop	F	ACAGAGGTCACACGCACATC	Xbp1	F	GAGCAGCAAGTGGTGGATTT
	F	CTTCCGGAGAGACAGACAGG		F	CTCTGGGGAAGGACATTTGA
c-Myc	R	CAACGTCTTGGAACGTCGTCAG A	Dnmt1	R	GCTGCTACCAAGGACTAG
	F	TCGTCTGCTTGAATGGACAG		F	ACAAGACTTCAAATGATGGG
Cycin D1	R	GCGTACCCTGACACCAATCT	Dnmt3a	R	ACTTGGAGAAGCGGAGTGAA
	F	ATCTCCTTCTGCACGCACTT		F	CTGTTCTTTGCCCTCTCCTG
Ezh2	R	TGTGGAGTTGGTAAATGC	Dnmt3b	R	GATGCTATTGTGAATGTG
	F	TTTCTTCTCTTTCATCTGGAT		F	AGGAAGACTTAAACCATAA
Grbarab	R	CAGCAGGAGGGGTAATGGTA	Eed5	R	GGGCACAGAGATGAAGT
	F	CCAATGTCAATCCCTTCCAC		F	CCAGGTTTCCAGCATAC
Grp78	F	TCTGGTGATCAGGATACAGGTG	Suz12	F	GAAGTGGAGCAGCAGAGAA
	R	TTCAGCTGTCACTCGGAGAATA		R	CTACAAACAGCATACAGGCA
Figla	F	ACAGAGCAGGAAGCCCAGTA	Lxh8	F	CAGTTCGCTCAGGACAACAA
	R	ACTCGCACAGCTGGTAGGTT		R	AGCCATTTCTTCCAACATGG
Foxo3a	F	GATGATGATGGACCCCTGTC	Nobox	F	ACAAACGCCATGAGATTTCC
	R	TCTTGGCGGTATATGGGAAG		R	AACAGGGCCAGGTTCTAGGT

Supplementary Table 3: Primers used for quantitative real-time polymerase chain reaction to quantify mitochondrial DNA copy number and functional regulation

	Gene symbol		Primer		Gene symbol		Primer
Mitochondrial DNA copy number	mCytb	F	ATTGACCTACCTGCCCCATC	Mitochondrial activity	Rhot2	F	CAGTTACCCGCGAGAAGAAG
		R	CTCGTCCGACATGAAGGAAT			R	GGCTGTCAGCATTCACTTCA
	Actin	F	TCGCCATGGATGACGATA		Cytochrome C	F	TTCCACAACCCTCATGTGAA
		R	CACGATGGAGGGGAATACAG			R	TAAGGGTCCAAAACCAGTGC
Mitochondrial activity	Mpv17	F	TCGGAGGCTGGTACAAAGTT		Atp5b	F	TGGGAAAATCGGACTCTTTG
		R	ATTGTCCTGGGCTGACATTC			R	AGTAACCACCATGGGCTTTG
	Cbr1	F	AGTGGTGAATGTGTCCAGCA		Atp synthase	F	GCCCTCGGTAATGCTATTGA
		R	CAGGACTGTCACCCCAATCT			R	CACAGAGATTCGGGGGATAA
	Imp1	F	GGCATCCAAAGAGGTGACAT		Sco1	F	AGTGGCCTTGAAAGAAAGCA
		R	ACATGACCTGTTGGCACGTA			R	GTAGCCTGCCCTTGCTATTG
	Mtfp1	F	GCTGTGGTGTGGTTGAGCTA		Sco2	F	CCTTCGCTGAACTTGTCCTC
		R	ACACAGACGGTTGATGGTGA			R	CCCTAGAGCCAGTAGCATCG

Supplementary Table 4: Primers used for quantitative real-time polymerase chain reaction for placenta analysis

Gene		Primer	Gene		Primer
Mash2	F	AGTTCCCAGCACCCATGTAG	Slc22a3	F	CATTTGCGCCAAATTTTTCT
	R	GGGTGGTTTGCTTGCAGTAT		R	TCACGATCACGAAGCAAGTC
Cdx2	F	TGTGATTGGAGGTTAAAGTG	Slc40a1	F	GTCATCCTCTGCGGAATCAT
	R	CCCAAAACAAACCTCACCAT		R	AAGGACCCATCCATGGTACA
Hand1	F	TGGACGTCTGAACCCTTCTC	Slc2a3	F	CGCCAATGGCTTTTAAGTGT
	R	AAGGCTGGAGATGACACGAA		R	CCCCTTCCCTCCCAATATAA
Gys1	F	GGGACACTGTGCATTGTTTG	Ig-DMR	F	TATATATGGATGTATTGTAATATAGGTTAG
	R	CCGATTCGTCTAATGGTGCT		R	CACTTTTACTATAAAACACTTAACTC
Gcm1	F	CGATGTGAAACTGCCTCAGA	Lit1-DMR	F	TAAGGTGAGTGGTTTAGGAT
	R	TGTCGTCCGAGCTGTAGATG		R	CCACTATAAACCCACACATA
Prl8a8	F	CCTCAGTGATGGAACTGCAA	Meg1-DMR	F	AAGTTTGTTTTGTTGAGTTGGTTTT
	R	GAAGCTTTCTCCCACAGCAG		R	AATATAATTCACCACCCATTCCTAA
Gjb3	F	CTTCTTCCCCATCTCCAACA	Peg3-DMR	F	GGTGGAGATAGGTTTGTTGTATAG
	R	AGGCTACATGCAGGATGACC		R	AAAACCAAATACAATCCAAAATC
Gbe1	F	CTCCTGGAGATTGACCCGTA	Peg10-DMR	F	TGGAAAGTTGTAGGAGAGTAATTAAA
	F	TCGATCCCACCTTCATTCTC		F	CACAAACTATTACTAAACACCCATTC
Atp2a3	R	TGACCCCCAGTAACAAAAGC	Rafg1-DMR	R	AGTTGGGGAGGTGTGTTATATTAGA
	F	GAGGAGATGCAGGAGAGTGG		F	AAACCAAAATATCAATCCTAACCTC

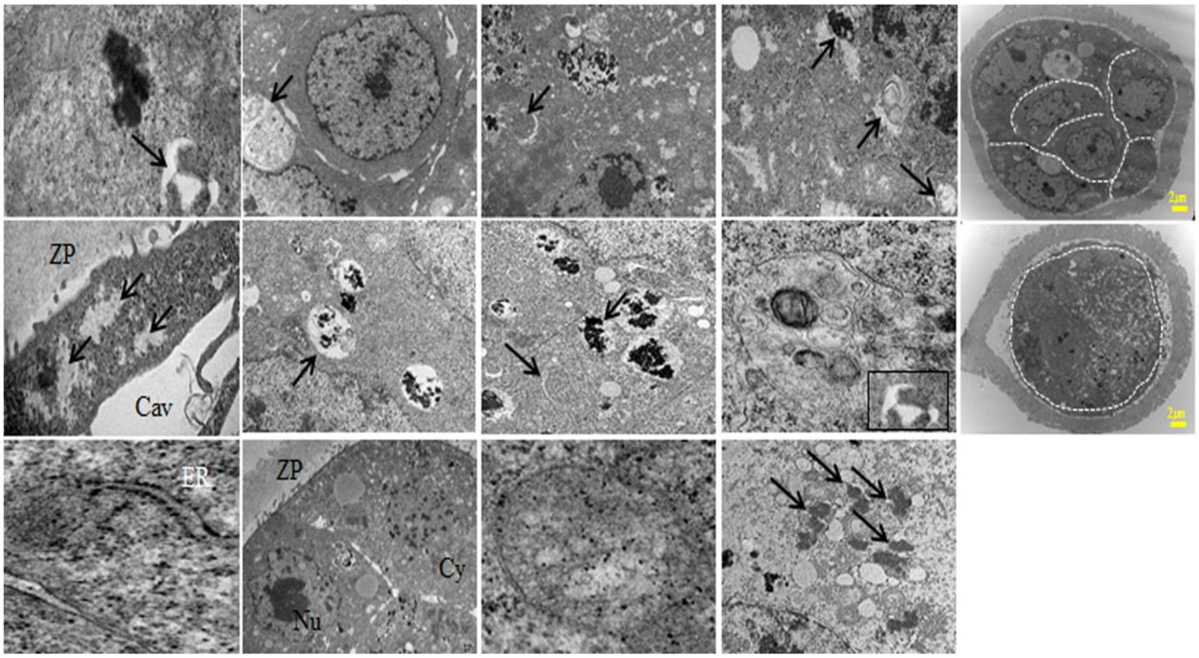

Supplementary Figure 1: The effect of rapamycin on CSNPs-induced autophagy in blastocysts. Morula-stage embryos were cultured with CSNPs. CSNPs-treated embryos exhibited various types of structural and cellular damages related to autophagy.

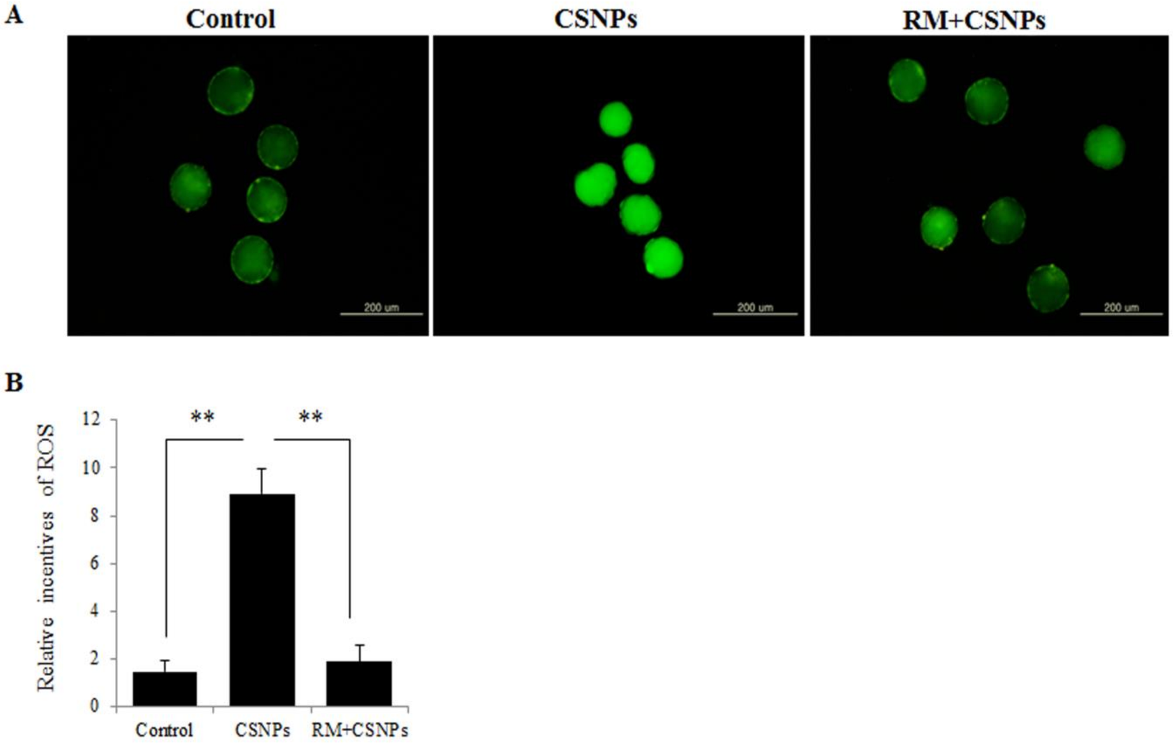

Supplementary Figure 2: Determination of ROS signals. (A). Levels of cytoplasmic ROS generation were measured (green color) after morula stage embryos were cultured either in CSNPs or rapamycin +CSNPs for 24h. (B) Statistical analysis of ROS intensity. RM indicates rapamycin.

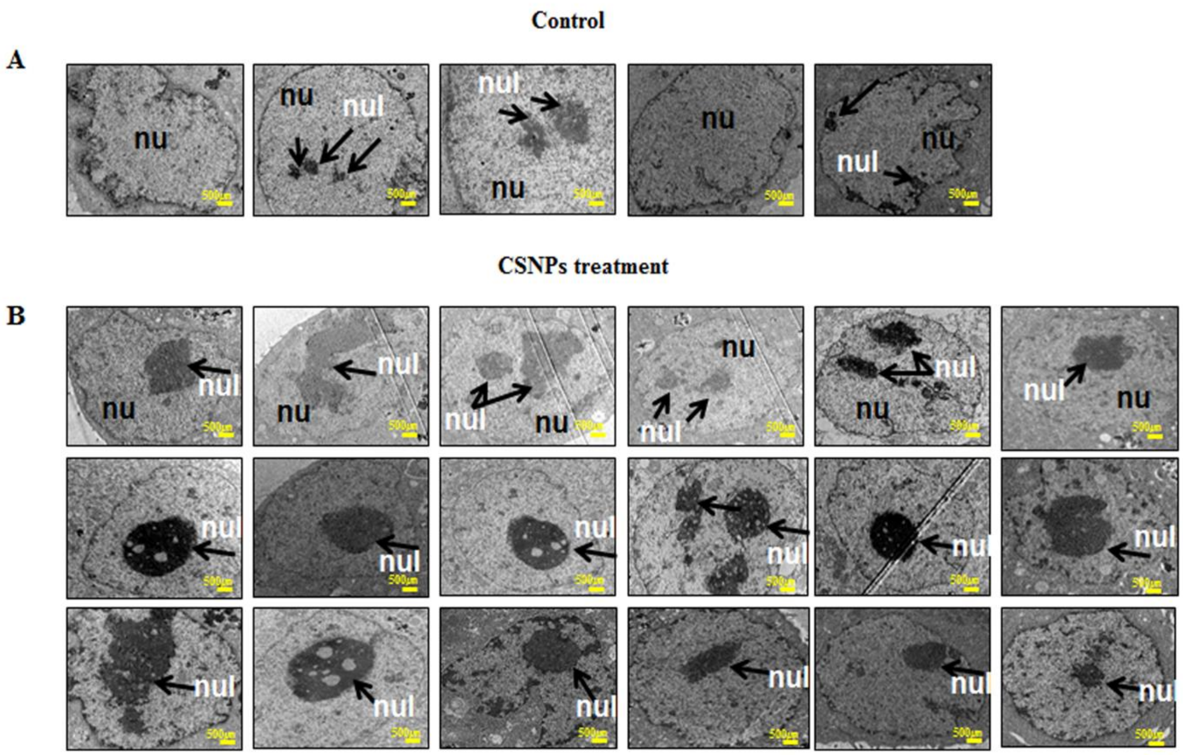

Supplementary Figure 3: Ultrastructure of heterochromatin condensation. (A) Control (B) CSNPs-treated embryos.

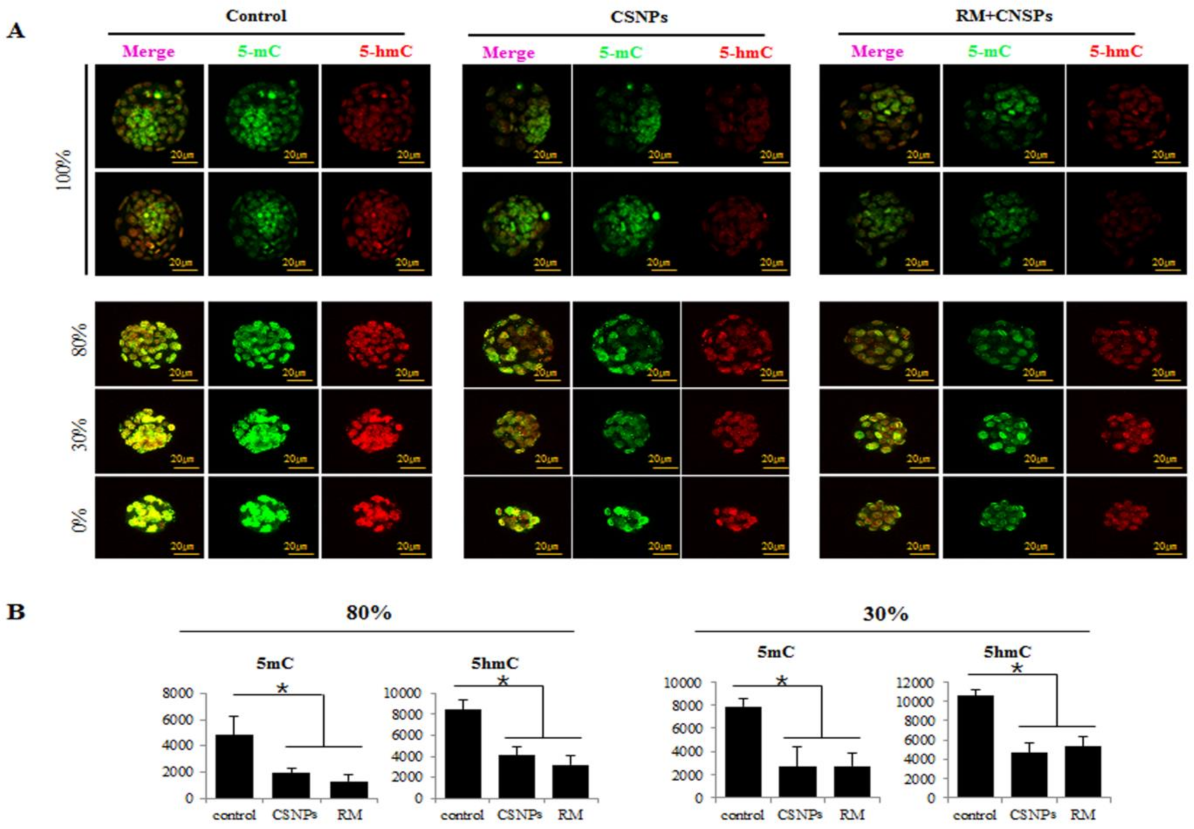

Supplementary Figure 4: Comparison of 5-hmC and 5-mC signal intensity in blastocyst with different cavity size. (A) 5-hmC and 5-mC staining patterns of the control, the CSNPs- and rapamycin+CSNPs-treated group. (B) Measurement of the fluorescence intensities of 5-hmC and 5-mC signals. RM indicates rapamycin.

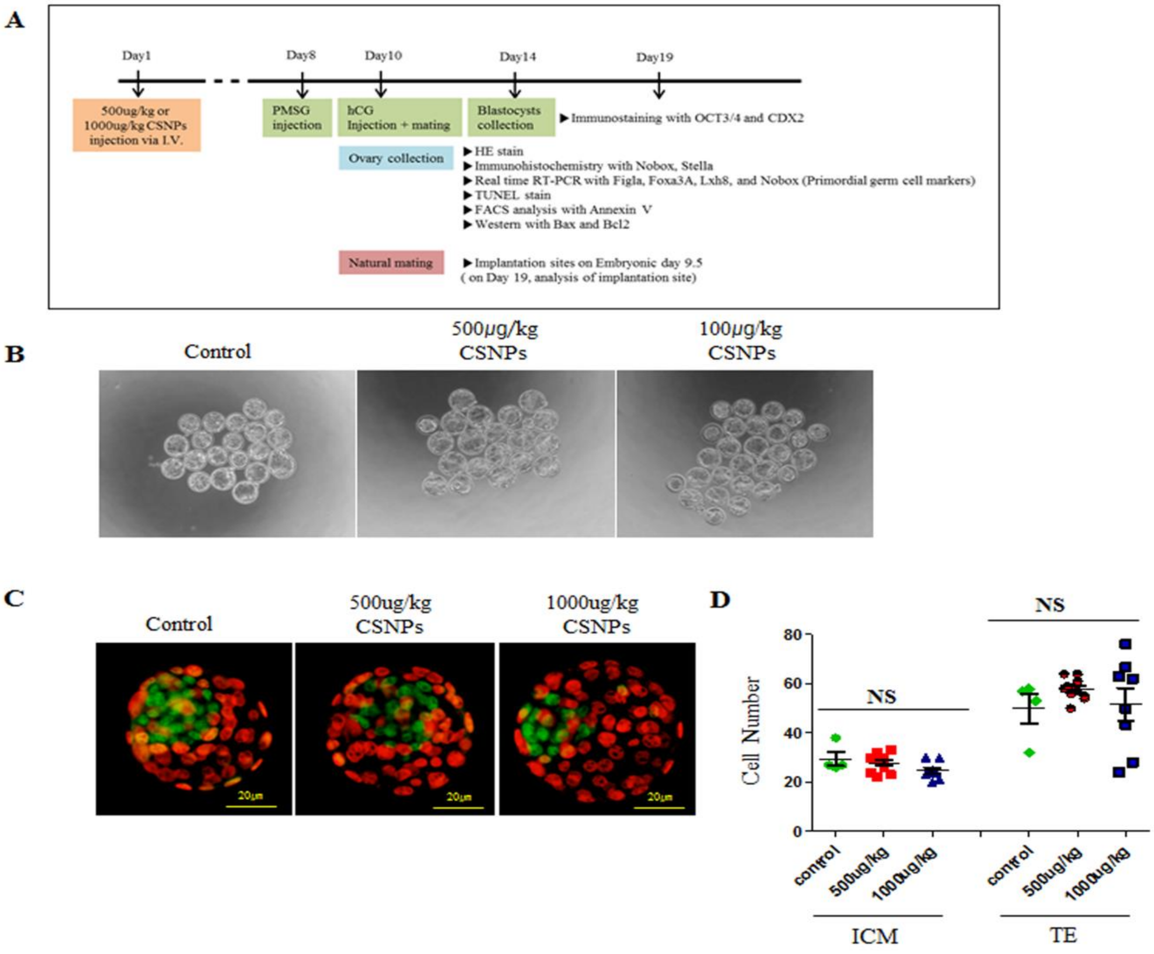

Supplementary Figure 5: Quality analysis of *in vivo* blastocyst embryos recovered from CSNPs-injected female mice. At 10 days after CSNPs injection, female mice are mated with fertile male mice and checked vaginal plugs at next morning. Three days later, blastocyst embryos were recovered from Control and CSNPs-treated female mice. (A) Time schedule for *in vivo* studies. (B) Microscopy picture of blastocyst stage embryos. (C) Cdx2 (red color) and Oct3/4 (green color) staining using CDX2 and OCT3/4 antibodies indicate (TE) and inner cell mass (ICM), respectively. (D) Statistical analysis of TE and ICM cell numbers. There is no difference between control and CSNPs-injected blastocyst stage embryos.

A

	No. of embryos transferred	No. of pregnancy	No. of offspring
Control	20	4	30
CSNPs	20	6	20
RM+CSNPs	20	6	28

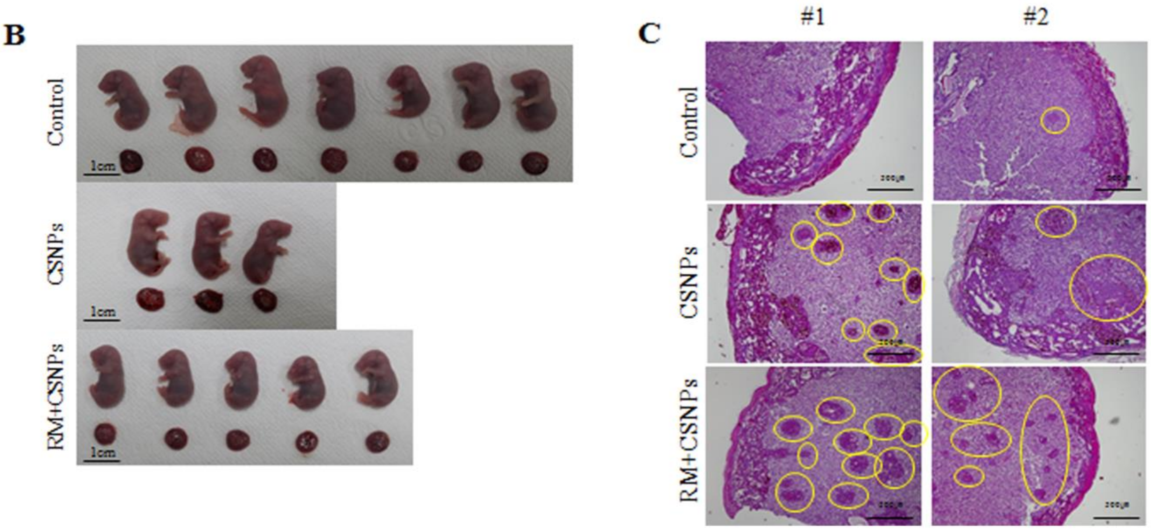

Supplementary Figure 6: Fertility test in CSNPs- or rapamycin+CSNPs treated embryos. Morula-stage embryos were cultured with CSNPs or rapamycin+CSNPs for 24h. At day 20 after embryo transfer, each offspring were recovered from the uteruses of recipients treated with CSNPs or both rapamycin and CSNPs. (A) Embryo transfer data in control, CSNPs, rapamycin+CSNPs treated groups. (B) A representative picture of offspring and placenta. (C) PAS staining of placentas in control, CSNPs, rapamycin+CSNPs treated groups. Of note, control shows normal spongiotrophoblast areas, whereas CSNPs-treated groups show mis-location of spongiotrophoblast. RM indicates rapamycin.

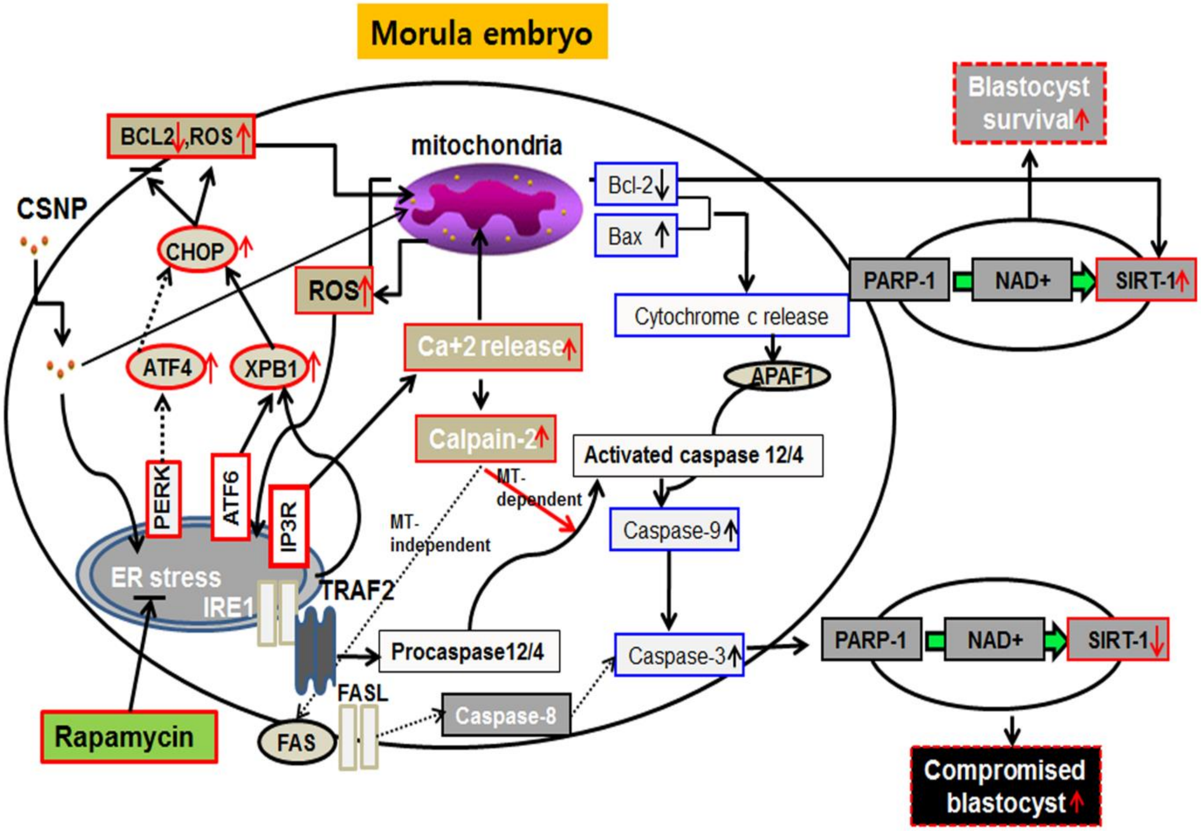

Supplementary Figure 7: The hypothetical mechanisms for explaining CSNPs-induced toxic effects in preimplantation mouse embryos.
